# Double-stranded RNA induces inflammation via the NF-κB pathway and inflammasome activation in the outer root sheath cells of hair follicles

**DOI:** 10.1038/srep44127

**Published:** 2017-03-07

**Authors:** Jung-Min Shin, Dae-Kyoung Choi, Kyung-Cheol Sohn, Soo-Yeon Kim, Jeong Min Ha, Young Ho Lee, Myung Im, Young-Joon Seo, Chang Deok Kim, Jeung-Hoon Lee, Young Lee

**Affiliations:** 1 Department of Dermatology, School of Medicine, Chungnam National University, Daejeon, Korea; 2 Department of Anatomy, School of Medicine, Chungnam National University, Daejeon, Korea

## Abstract

Alopecia areata (AA), a chronic, relapsing, hair-loss disorder, is considered to be a T cell-mediated autoimmune disease. It affects approximately 1.7% of the population, but its precise pathogenesis remains to be elucidated. Despite the recent attention focused on the roles of inflammasomes in the pathogenesis of autoinflammatory diseases, little is known about inflammasome activation in AA. Thus, in this study, we investigated the pattern of NLRP3 inflammasome activation in the outer root sheath (ORS) cells of hair follicles. We found that interleukin (IL)-1β and caspase-1 expression was increased in hair follicle remnants and inflammatory cells of AA tissue specimens. After stimulation of ORS cells with the double-stranded (ds)RNA mimic polyinosinic:polycytidylic acid (poly[I:C]), the activation of caspase-1 and secretion of IL-1β were enhanced. Moreover, NLRP3 knockdown decreased this poly(I:C)-induced IL-1β production. Finally, we found that high-mobility group box 1 (HMGB1) translocated from the nucleus to the cytosol and was secreted into the extracellular space by inflammasome activation. Taken together, these findings suggest that ORS cells are important immunocompetent cells that induce NLRP3 inflammasomes. In addition, dsRNA-induced IL-1β and HMGB1 secretion from ORS cells may contribute to clarifying the pathogenesis and therapeutic targets of AA.

Alopecia areata (AA) is the most common cause of inflammation-induced hair loss, which mainly occurs via T-cell-mediated attack of anagen-stage hair follicles with the collapse of immune privilege^1^,^2^. The pathogenesis of AA is poorly understood, but there is emerging evidence that both innate and acquired immunity are involved^3^. AA is associated with various autoimmune disorders such as autoimmune thyroiditis, type I diabetes, and vitiligo, giving considerable support to the assertion that it is a tissue-specific autoimmune disease^4^,^5^. In addition, recent genetic studies of AA identified multiple genes that control acquired and innate immunity, and suggested that several factors act together to induce and promote immune dysregulation in the pathogenesis of this condition^3^,^6^.

The innate immune response is the first line of defense, providing an appropriate defense mechanism for maintaining the integrity and homeostasis of an organ. It is also a sophisticated system for sensing signals of danger, such as pathogens, host-derived cellular stress, dead cells, or irritants^7^. Dysregulation of the innate immune system can cause insufficient inflammation, which can result in persistent infection, prolonged inflammation, and chronic systemic inflammatory diseases^8^. The inflammasome is a large macromolecular complex that is an important component of the innate immune response. In contrast to toll-like receptors (TLRs), it is not only assembled upon the sensing of pathogen-associated molecular patterns (PAMPs), but also by contacting endogenous molecules released by damaged or injured cells, termed damage-associated molecular patterns^9^,^10^.

Recent studies have shown the presence of genetic mutations in inflammasome pathways in cases of autoinflammatory disease, suggesting that there is an integral and essential relationship between adaptive responses and innate immunity in autoinflammatory disease^11^,^12^,^13^. However, our understanding of the involvement of inflammasome activation in AA is limited, although several papers have described the clinical significance of the interleukin (IL)-1 family in AA susceptibility and severity^14^,^15^,^16^. Previous report showed the increased expression of IL-1β in lesional skin of AA and suggested its role in hair growth cycle^14^. IL-1 is an active pro-inflammatory cytokine secreted upon the activation of inflammasomes. Inflammasomes are highly expressed in inflammatory cells such as macrophages, but several lines of evidence have also shown that they are also expressed in various other cells such as hepatocytes, hematopoietic cells, lung cancer cells, and keratinocytes^9^,^17^. Keratinocytes are an important source of IL-1 in the skin, and these non-professional immune cells are immunologically active and involved in different auto-inflammatory skin diseases such as psoriasis and vitiligo^18^,^19^,^20^,^21^.

The outer root sheath (ORS) of hair follicles is a multilayered tissue that surrounds the hair fiber and inner root sheath; it contributes to the generation of hair follicles and the epidermis. AA is associated with apoptosis of ORS cells, which induces the catagen phase in hair follicles, cell degeneration, and increased levels of apoptosis and necrosis in hair follicles^22^. To determine the role of the inflammasome in the pathogenesis of AA, we investigated whether polyinosinic:polycytidylic acid (poly[I:C]), a synthetic double-stranded RNA (dsRNA), triggers IL-1β-mediated inflammatory responses via the NF-κB pathway and inflammasome activation in human ORS cells of hair follicles.

## Results

### Expression of inflammasome components and IL-1β is increased in AA

**Figure 1:**
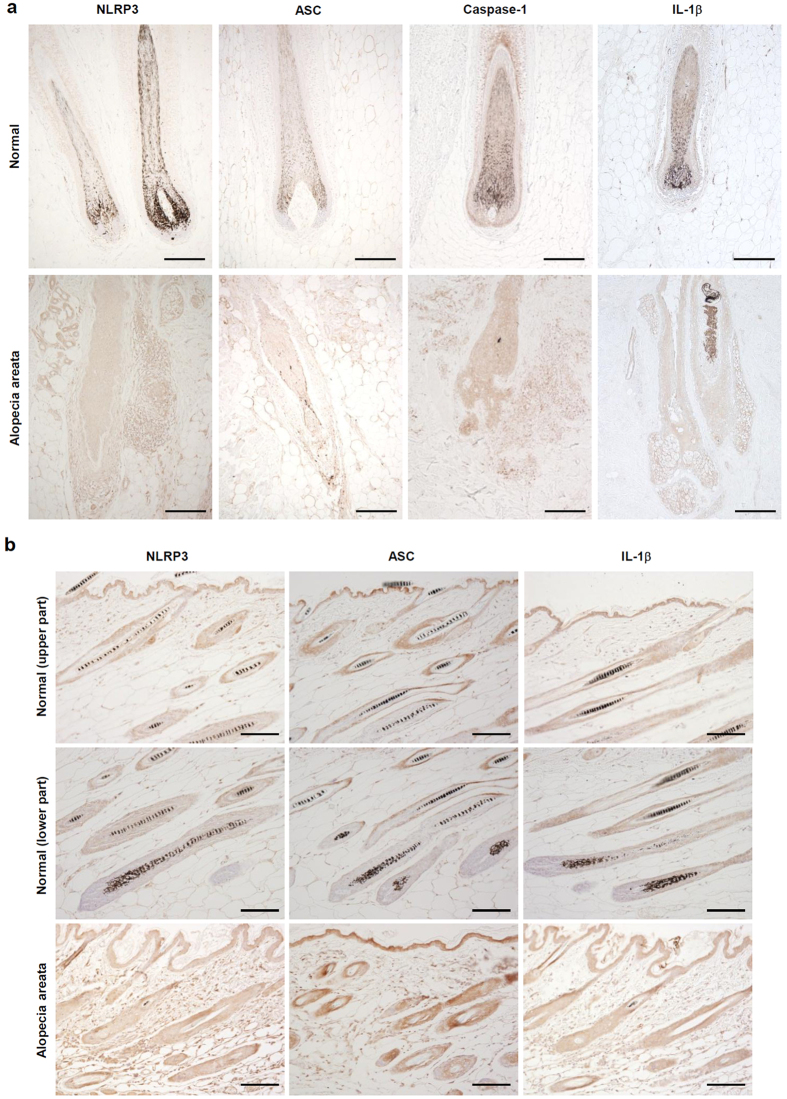
Expression of inflammasome components and interleukin (IL)-1β in normal and alopecia areata-affected scalp tissues. (**a**) Paraffin-embedded normal scalp skin and alopecia areata lesions were immunohistochemically stained with anti-NLRP3, ASC, caspase-1 and IL-1β. (**b**) Skin sections from normal and spontaneous AA of C3H/HeJ mice were immunohistochemically stained with anti-NLRP3, ASC and IL-1β. Bars = 100 μm.

To investigate whether inflammasome activation is involved in the pathogenesis of AA, we examined the expression of NLRP3, ASC, caspase-1, and IL-1β in the skin lesions of patients with AA compared to that in normal scalp skin. Immunohistochemical staining showed that NRLP3, ASC, and caspase-1 were significantly increased in the ORS of and infiltrated inflammatory cells around hair follicles in AA. IL-1β was also highly expressed in the ORS of hair follicles in AA (Fig. 1a). To confirm the inflammasome activation in AA, we used the C3H/HeJ AA mice model that spontaneously develops AA with considerable similarity to human AA. In normal C3H/HeJ mice, NLRP3, ASC, and IL-1β were expressed in the interfollicular epidermis and upper part of hair follicles, but the expressions were scarce in the lower part of hair follicles, which are mostly damaged during inflammation in AA. In the lesional skin of C3H/HeJ AA mice, NLRP3, ASC, and IL-1β were highly expressed in the ORS of hair follicles and inflammatory infiltrates (Fig. 1b). These findings suggest that inflammasome activation in the ORS of hair follicles may be linked to the pathogenesis of AA.

### Poly(I:C) induces pyroptosis and NLRP3 inflammasome activation in ORS cells

**Figure 2:**
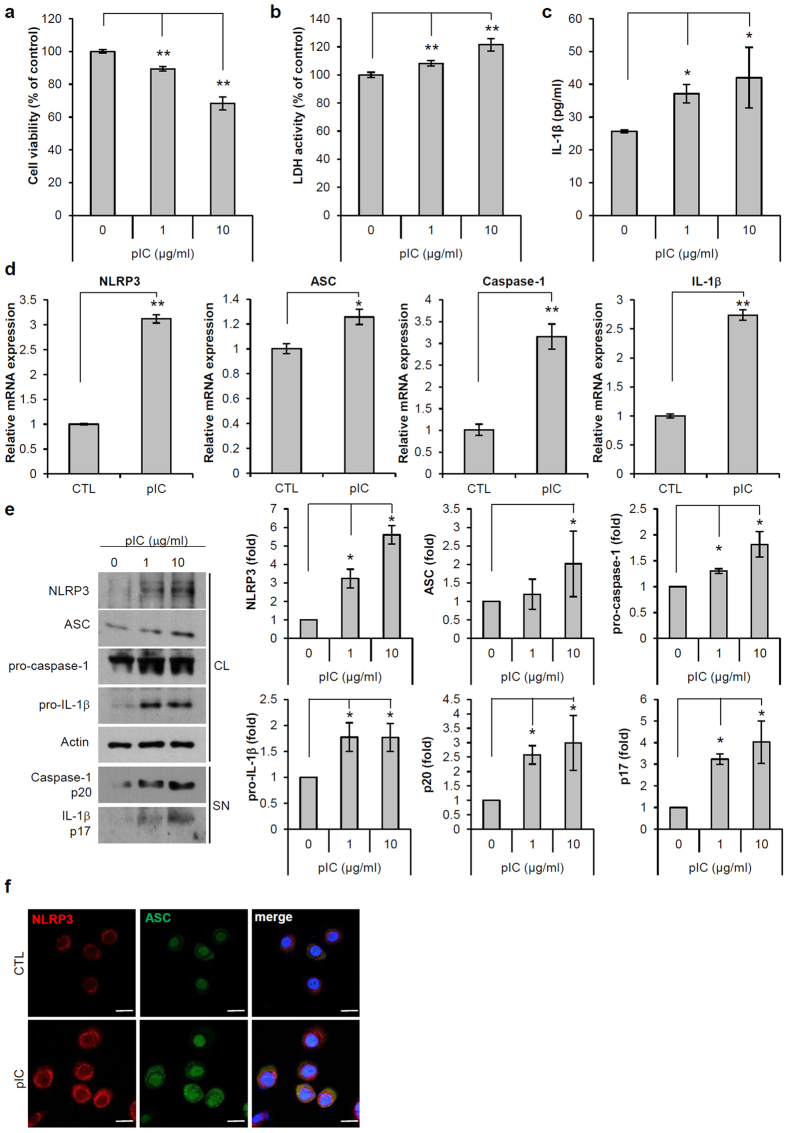
Polyinosinic:polycytidylic acid (poly[I:C]) induces pyroptosis and NLRP3 inflammasome activation in outer root sheath (ORS) cells. ORS cells were treated with poly(I:C) at the indicated concentrations for 24 h. (**a**) Cell viability was measured by 3-(4,5-dimethylthiazol-2-yl)-2,5-diphenyltetrazolium bromide (MTT) assay. (**b**) Pyroptosis was determined by lactate dehydrogenase activity. (**c**) IL-1β secretion in supernatants (SN) was assessed using enzyme-linked immunosorbent assay (ELISA). (**d**) Relative mRNA expression of IL-1β, NLRP3, ASC, and caspase-1 was determined by quantitative reverse transcription polymerase chain reaction. (**e**) Expression levels of NLRP3, pro-caspase-1, and IL-1β in the cell lysates (CL) were assessed by Western blotting. Actin was used as a loading control. Expression levels of active caspase-1 p20 subunit and mature IL-1β p17 in SN were determined by Western blotting. (**f**) Co-localization of NLRP3 (red) and ASC (green) was detected by immunofluorescence staining. Nuclei were counterstained with 4,6-diamidino-2-phenylindole (blue). Bars = 20 μm. Data are presented as mean ± standard error of the mean (SEM). Data were analyzed by one-way ANOVA (**a**–**c**,**e**) or Student’s *t*-test (**d**) (n = 3, *p < 0.05, **p < 0.01). Cropped blots were used in this figure and full-length blots are presented in Supplementary Figure 4.

Poly(I:C) is a synthetic dsRNA analogue that interacts with TLR3 and activates innate immunity^23^. We first examined the expression of TLR3 in humans and in a mouse AA model (Supplementary Fig. S1). In the human control, TLR3 was mainly expressed in the interfollicular epidermis and upper part of hair follicles. However, in AA, TLR3 expression was increased in the lower part and bulb of hair follicles. The results were similar in the mouse AA model. We determined its effects in hair follicle cells including ORS and dermal papilla (DP) cells. When poly(I:C) was applied at a range of doses, it induced cell death and lactate dehydrogenase release in ORS cells in a dose-dependent manner, but had no effect on DP cells (Fig. 2a,b, Supplementary Fig. S2). Poly(I:C) also increased the secretion of IL-1β from ORS cells (Fig. 2c). We confirmed that poly(I:C) upregulated the mRNA levels of inflammasome components such as NLRP3, ASC and caspase-1, and also significantly increased IL-1β mRNA levels (Fig. 2d). Significant increases of NLRP3, pro-caspase-1 and pro-IL-1β protein expression by poly(I:C) were also detected by Western blot analysis of cell lysates. Next, we evaluated whether poly(I:C) treatment induced inflammasome activation, and found that the secretion of active caspase-1 p20 and IL-1β p17 was significantly increased in cultured medium for ORS cells (Fig. 2e). The co-localization of NLRP3 and ASC was also increased by poly(I:C) treatment, indicating the assembly of these inflammasome molecules (Fig. 2f). These results indicate that poly(I:C) can act as both a priming signal and an activating signal for inflammasome activation in ORS cells.

### Poly(I:C)-induced IL-1β secretion depends on inflammatory caspases and NLRP3 inflammasome activation

**Figure 3:**
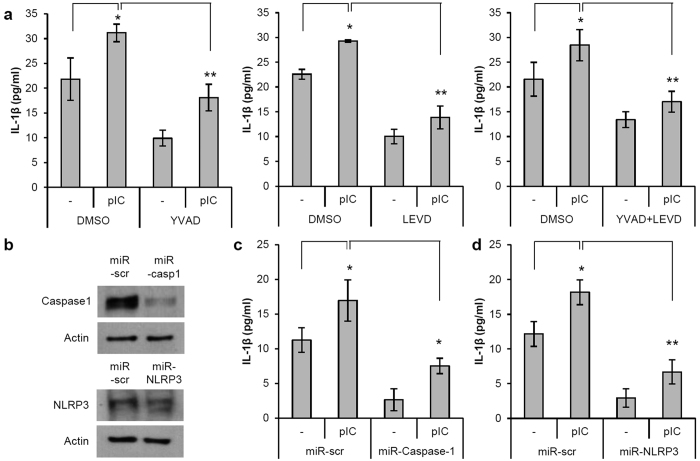
Poly(I:C)-induced IL-1β secretion depends on NLRP3 inflammasome activation in ORS cells. (**a**) ORS cells were pretreated with specific caspase-1 and/or caspase-4 inhibitors for 2 h and then stimulated with poly(I:C) (10 μg/mL) for 24 h. IL-1β secretion in supernatants was assessed using ELISA. (**b**) ORS cells were transduced with adenoviruses expressing caspase-1- and NLRP3-specific microRNA (miR) and cultured for 3 days. Scrambled microRNA (miR-scr) was used as a control. Knockdown of caspase-1 and NLRP3 was determined by Western blotting. (**c**,**d**) After the knockdown of caspase-1 or NLRP3 in ORS cells, cells were further treated with poly(I:C) (10 μg/mL) for 24 h. Supernatants were analyzed for IL-1β by ELISA. Data are presented as means ± SEM. Data were analyzed by one-way ANOVA (n = 3, *p < 0.05, **p < 0.01). Cropped blots were used in this figure and full-length blots are presented in Supplementary Figure 5.

Inflammatory caspases, such as caspase-1 and caspase-4, regulate inflammasome activation^9^,^10^. To determine whether the activation of inflammatory caspases is required for poly(I:C)-induced IL-1β secretion in ORS cells, specific inhibitors were used to block their activity. Pretreatment with caspase-1 and/or caspase-4 inhibitors significantly blocked poly(I:C)-induced IL-1β secretion from ORS cells (Fig. 3a). To further evaluate the functional role of caspase-1 and NLRP3 in IL-1β secretion, we generated recombinant adenoviruses expressing microRNA specific for caspase-1 and NLRP3 to knock down their expression (Fig. 3b). Knockdown of caspase-1 and NLRP3 significantly inhibited poly(I:C)-induced IL-1β secretion (Fig. 3c,d). These findings indicate that NLRP3 inflammasome activation is required for IL-1β secretion by poly(I:C) treatment.

### Poly(I:C) activates IL-1β secretion by regulating the NF-κB signaling pathway

**Figure 4:**
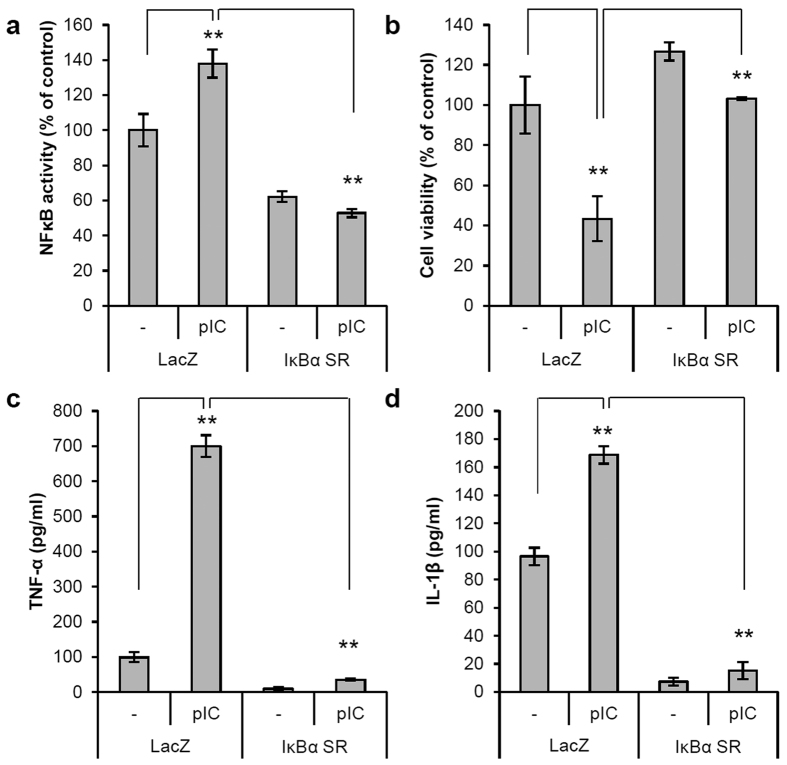
Poly(I:C) induces IL-1β secretion by activating the NF-κB signaling pathway. (**a**) An NF-κB-luciferase reporter adenovirus was co-transduced with adenoviruses expressing LacZ (control) or IκBα super-repressor (SR) and cultured for 2 days. Then, cells were treated with poly(I:C) (10 μg/mL) for 4 h. Cells were lysed and assayed for luciferase activity. (**b**) After the overexpression of LacZ (control) or IκBα super-repressor (SR), cells were treated with poly(I:C) (10 μg/mL) for 24 h. Cell viability was measured by MTT assay. (**c**,**d**) Supernatants were also analyzed for IL-1β and tumor necrosis factor-α by ELISA. Data are presented as means ± SEM. Data were analyzed by one-way ANOVA (n = 3, **p < 0.01).

Poly(I:C) is a well-known TLR3 ligand that activates the NF-κB signaling pathway. Thus, we evaluated whether poly(I:C) regulates IL-1β secretion through the NF-κB signaling pathway in ORS cells. To inhibit NF-κB activation, we introduced a recombinant adenovirus expressing the IκBα super-repressor, which lacks the phosphorylation sites necessary for proteasomal degradation. Overexpression of this IκBα super-repressor attenuated NF-κB activity and cell death by poly(I:C) treatment (Fig. 4a,b). Inhibition of NF-κB by the IκBα super-repressor significantly inhibited poly(I:C)-induced tumor necrosis factor-α and IL-1β secretion (Fig. 4c,d). These findings suggest that poly(I:C) induces cell death and IL-1β secretion via the NF-κB signaling pathway.

### Poly(I:C) induces the translocation and release of HMGB1 from ORS cells

**Figure 5:**
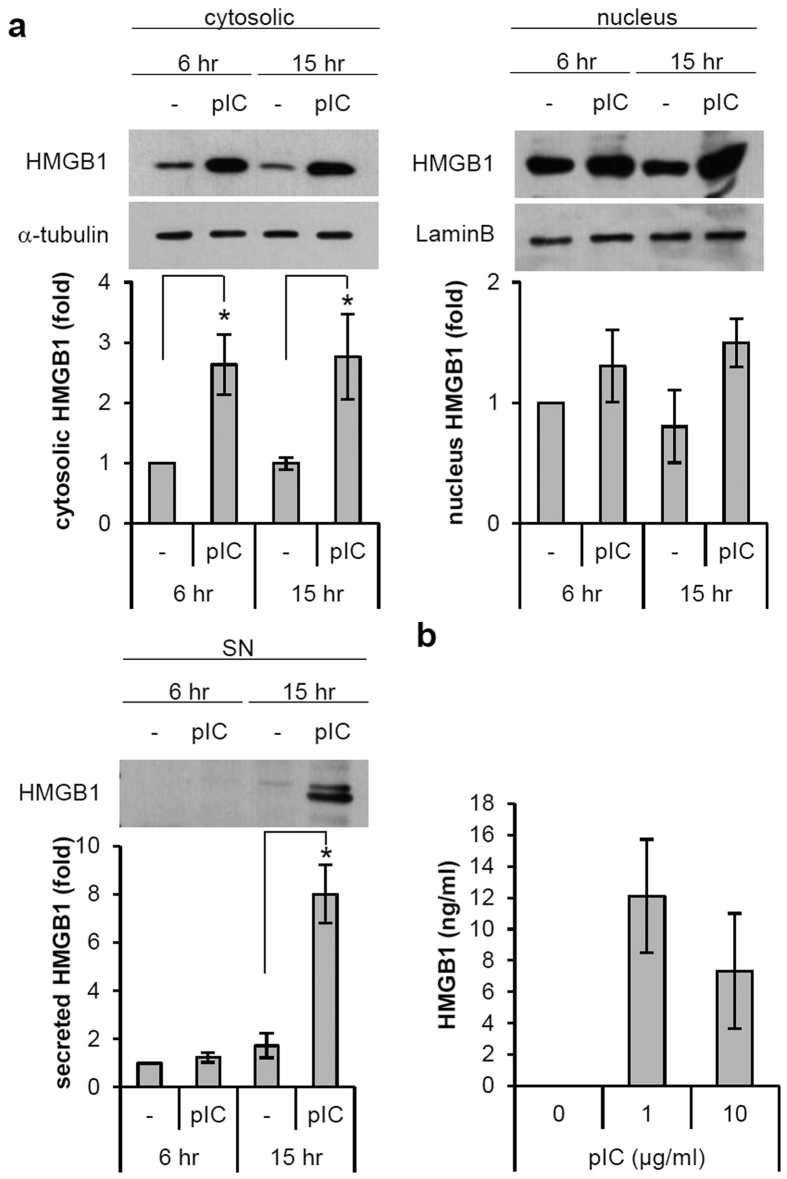
Poly(I:C) regulates the translocation and release of HMGB1 in ORS cells. (**a**) ORS cells were treated with poly(I:C) (10 μg/mL) for the indicated times. Cell extracts were fractionated, and the expression level of HMGB1 was assessed by Western blotting. α-tubulin and laminB were used for determination of the cytoplasmic and nuclear fractions, respectively. The release of HMGB1 in supernatants (SN) was also assessed by Western blotting. (**b**) ORS cells were treated with poly(I:C) at the indicated concentrations for 24 h. Supernatants were analyzed for HMGB1 by ELISA. Data are presented as means ± SEM (n = 3). Data were analyzed by one-way ANOVA (n = 3, *p < 0.05). Cropped blots were used in this figure and full-length blots are presented in Supplementary Figure 6.

HMGB1 acts as a pro-inflammatory mediator and is elevated in several autoimmune diseases^24^,^25^,^26^. We previously reported that HMGB1 is highly expressed in the lesional skin of patients with AA^27^. In this study, we demonstrated that the dsRNA mimic poly(I:C) regulates the translocation and secretion of HMGB1 from ORS cells. When ORS cells were treated with poly(I:C), the cytoplasmic (Fig. 5a) and secreted levels (Fig. 5b) of HMGB1 markedly increased. These findings suggest that hair follicle ORS cells can serve as another source of HMGB1 in AA, in addition to activated macrophages.

## Discussion

Inflammasomes act as signaling platforms that can respond to a plethora of microbial products, as well as endogenous host products associated with cellular stress and damage, and play a vital role in innate immunity^9^,^10^. These multi-protein complexes are composed of a NOD-like receptor (NLR)/and an AIM-like receptor, the adapter molecule apoptosis-associated speck-like protein that contains a caspase recruitment domain (ASC) and pro-caspase-1. ASC is a scaffold protein associated with an NLRP in many inflammasomes. NLRP3 is one of the best-characterized NLRPs^28^. The activation of inflammasomes leads to caspase-1 activation and secretion of the inflammatory cytokines IL-1 and IL-18, resulting in inflammation *in vivo*^29^,^30^. Previous studies revealed that inflammasomes are associated with several autoinflammatory and autoimmune diseases such as rheumatoid arthritis, pyogenic arthritis, systemic sclerosis, and systemic lupus erythematosus^12^,^13^,^31^.

The aberrant activity of cytokines and inflammasomes is involved in the pathogenesis of dermatological diseases such as acne, pyoderma gangrenosum, psoriasis, vitiligo, and contact dermatitis^32^,^33^. In addition, genetic polymorphisms in inflammasome components were found in patients with psoriasis- and vitiligo-associated autoimmune diseases^34^,^35^. Although there is no report on whether inflammasomes are related to the pathogenesis of AA, various pieces of evidence suggest the clinical significance of IL-1, which is the downstream factor of inflammasomes in AA. Previous studies have described IL-1 as a crucial mediator inducing cessation of hair growth *in vivo* and *in vitro* suggesting IL-1 signaling system is involved in catagen development. Also genetic analysis showed the significant association of the IL-1 family to AA susceptibility and severity^14^,^15^,^16^. In the current study, we provide evidence that human ORS cells contain the necessary elements to form NLRP3 inflammasomes and dsRNA upregulates inflammasome elements and induce IL-1β via NLRP3 inflammasome activation. These findings suggest that AA is an autoinflammatory diseased linked to the hyperactivation of the innate immune system. However, as IL-1β is known to be a potent inhibitor of human hair growth *in vitro* and increased during catagen period in murine skin, the increased expression of IL-1β in AA lesion in our study cannot completely exclude the possibility of variations in the hair growth^14^,^16^.

The NLRP3 inflammasome is primed before activation in two distinct steps. The first step is NF-κB activation through TLR, which increases the expression of NLRP3 and pro-IL-1β. The second step requires a distinct signal to activate NLRP3, leading to the formation of NLRP3 inflammasome complexes. Pathogens, PAMPs, danger-associated molecular patterns, and environmental irritants can activate NLRP3^10^. However, recent reports have suggested that NLRP3 inflammasomes can also be activated by endogenous host-derived molecules^36^,^37^,^38^. Inefficient clearance of cellular debris in SLE results in the elevation of reactive oxygen species and mediates NLRP3 activation, and the accumulation of cytosolic self-DNA in autoimmunity triggers AIM2 inflammasome-mediated IL-1β production^37^. In this study, we applied treatment using poly(I:C), which is a synthetic dsRNA that activates the inflammasome in ORS cells via TLR3. The precise danger molecules that activate the inflammasome in ORS cells in AA remain unknown, but there are several possibilities. First, the insufficient clearance of cellular debris during the transition from the anagen to the catagen phase might induce inflammasome activation. Second, several reports have suggested that infectious agents such as viruses are potential inducers of AA^39^,^40^. The cellular debris and cytokines released by inflammatory cells during infection may induce inflammasome activation. However, both of these hypotheses are applicable to genetically susceptible patients with AA, which could explain the mechanism behind the frequent recurrence of AA lesions. Inflammasome activation mainly depends on TLR activation with downstream NF-κB signaling, which increases the expression of NLRP3 and pro-IL-1β. A few molecules, such as amyloid-β, can induce both NLRP3 priming through TLR activation and NLRP3 inflammasome activation^41^. In ORS cells, the knockdown of NF-κB signaling abolished poly(I:C)-induced inflammasome activation and IL-1β secretion, indicating that poly(I:C)-induced inflammasome activation in ORS cells is dependent on TLR3 and NF-κB signaling.

In this study, we also characterized the expression of HMGB1, which is released upon inflammasome activation. Previously, we investigated its expression in both the skin and serum samples of AA and its correlation with clinical markers associated with disease severity, suggesting its possible role in the pathogenesis of AA^27^. HMGB1 was originally identified as a nuclear DNA-binding protein, but during the inflammatory process, it can be secreted by cells and act as a proinflammatory cytokine^42^. An increase in HMGB1 has been reported in several autoimmune diseases such as rheumatoid arthritis, SLE, and systemic sclerosis^24^,^25^,^26^. Recent studies have revealed the novel mechanism underlying HMGB1 release during inflammation, and demonstrated that the inflammasome plays a critical role in mediating this release from immune cells^43^,^44^. Our study demonstrated the translocation of HMGB1 from the nucleus to the cytosol and its extracellular secretion after poly(I:C) treatment, which is consistent with the findings of previous studies that examined this issue in immune cells. The results revealed that ORS cells are non-professional immune cells that secrete proinflammatory cytokines such IL-1β and HMGB1.

This study provides the first evidence that human ORS cells of hair follicles constitutively express inflammasome proteins; and dsRNA triggers NLRP3 inflammasome activation, resulting in IL-1β and HMGB1 release from ORS cells. These findings indicate that the inflammasome-dependent activation and secretion of IL-1β is not restricted to macrophages and lymphocytes in AA; and suggest a functional role of hair follicle cells, particularly ORS, in innate immunity and the pathogenesis of AA. Our findings may provide new avenues for preventing the recurrence of AA or treating it by modulating the inflammasome activation pathway.

## Materials and Methods

### Cell culture and SV40 transformation

Human scalp tissues were obtained under the written informed consent of donors in accordance with the ethical committee approval process of the Institutional Review Board of Chungnam National University Hospital (IRB No. 1011-135, 2016-07-009). AA scalp tissues were obtained from 6 patients, from active AA lesions and normal scalp tissue, and ORS cells were obtained from occipital scalp skin that was the remnant of hair transplantation surgery, from 4 male individuals. The study was conducted in accordance with the Principles of the Declaration of Helsinki. Hair follicles were isolated from scalp specimens according to a previously reported method^45^. Hair follicles were incubated with 0.25% trypsin, 0.02% ethylenediaminetetraacetic acid (EDTA) in phosphate-buffered saline (PBS) for 10 min. Hair follicles were then vigorously pipetted to obtain single cell populations. The dissociated cells were rinsed in Dulbecco’s modified Eagle’s medium (HyClone, Logan, UT, USA) supplemented with 10% fetal bovine serum (Gibco, Grand Island, NY, USA), and centrifuged for 5 min at 200 g. ORS cells were then resuspended in keratinocyte-serum free medium (K-SFM) supplemented with epidermal growth factor (EGF) and bovine pituitary extract (Gibco) and seeded onto culture dish. Cultures were maintained at 37 °C in a humidified atmosphere of 5% CO_2_. For SV40 transformation, the retroviral vector pLXIN-SV40T was stably transfected into PT67 cells (Clontech Laboratories, Mountain View, CA), a recombinant retrovirus packaging cell line. Retrovirus-containing medium was collected, filtered through a 0.22-μm low protein binding filter (Millipore, Billerica, MA) and then transferred to primary cultured ORS and DP cells. After infection overnight, the retrovirus-containing medium was replaced with fresh medium and the cells were incubated for two additional days. SV40-transformed cells were selected in medium containing G418 (200 μg/ml) for 4 weeks, as reported previously.

### Mice

Female C3H/HeJ mice that spontaneously developed AA obtained from The Jackson Laboratory (Bar Harbor, Maine, USA). The mouse work was all reviewed and approved by Chungnam National University institutional animal care and use committee (IRB CNU-00733). All experiments were performed in accordance with institutional guidelines. These animals were followed until 15 months of age. A total of 30 female mice were observed for development of alopecia. Two mice developed clinical AA that was confirmed by histopathology.

### Reagents

Poly(I:C) was obtained from InvivoGen (San Diego, CA). Caspase-1 inhibitor (Z-YVAD-FMK) and caspase-4 inhibitor (Z-LEVD-FMK) were purchased from BioVision (Milpitas, CA). For immunohistochemical analysis and western blotting, we used the following specific antibodies: IL-1β and HMGB1 (Abcam, Cambridge, MA), caspase-1 (Cell Signaling Technology, Danvers, MA), NLRP3 and ASC (Adipogen, San Diego, CA), and actin, α-tubulin and laminB (Santa Cruz Biotechnologies, Santa Cruz, CA).

### Immunohistochemistry

Tissue samples were fixed with 10% formaldehyde, embedded in paraffin, and cut into 4-μm-thick sections. The sections were deparaffinized in xylene and then rehydrated using an alcohol series. The primary antibody was diluted 1:100 and was incubated at 4 °C for overnights. The sections were then incubated with secondary antibody at room temperature for 30 minutes. The sections were incubated with diaminobenzidine tetrachloride solution at room temperature for 1 minute and counterstained with Mayer’s hematoxylin. All human skin samples were obtained with the written informed consent of the donors and in accordance with the ethical committee approval process of the Institutional Review Board of Chungnam National University Hospital.

### MTT assay and LDH assay

Cells were treated with 5 mg/ml MTT (3-(4,5-dimethylthiazol-2-yl)-2,5-diphenyltetrazolium bromide) solution and were incubated for a further 30 min. The medium was removed and the resulting formazan crystal was solubilized in dimethylsulfoxide (DMSO). The optical density at 540 nm was determined using an ELISA reader. LDH activities were determined using Cytotoxicity detection kit (Roche, Penzberg, Germany), according to the recommended protocol.

### ELISA

Culture medium was collected, and secreted IL-1β, TNF-α and HMGB1 were determined using commercial ELISA kits. IL-1β and TNF-α kits were purchased from R&D Systems (Minneapolis, MN). HMGB1 kit was MyBioSource (San Diego, CA).

### Quantitative real-time polymerase chain reaction (qPCR)

Total RNAs were isolated using Easy-blue RNA extraction kit (Intron, Daejeon, Korea). Two μg of total RNAs were reverse transcribed with moloney-murine leukaemia virus (M-MLV) reverse transcriptase (RTase) (Elpis Biotech, Daejeon, Korea). Aliquots of RT mixture were amplified using SYBR Green real-time PCR master mix (Applied Biosystems, Waltham, MA). The following primers sequences were used: NLRP3, 5′-TCTGTGTGTGGGACTGAAGCA-3′ and 5′-CAGCTGACCAACCAGAGCTTCT-3′; ASC, 5′-GGCCGAGCCCACCAA-3′ and 5′-CAGGCTGGTGTGAAACTGAAGA-3′; Caspase-1, 5′-AAAAAATCTCACTGCTTCGGACAT-3′ and 5′-TCTGGGCGGTGTGCAAA-3′; IL-1β, 5′-TTAAAGCCCGCCTGACAG A-3′ and 5′-GCGAATGACAGAGGGTTTCTTAG-3′.

### Immnunofluorescence

Cells were grown on coverslips, fixed via incubation with 4% paraformaldehyde for 20 minutes, and permeabilized via incubation with 0.1% Triton X-100 in PBS for 10 minutes at room temperature. Cells were incubated overnight at 4 °C with anti-NLRP3 and anti-ASC antibodies (1:100) and then for 1hr at room temperature with Alexa Fluor dyes-conjugated secondary antibodies, and they were finally visualized under a confocal microscope (Leica Microsystems GmbH, Wetzlar, Germany).

### Western blot analysis

Cells were lysed in Proprep solution (Intron, Deajeon, Korea). Total protein was measured using a BCA protein assay kit (Pierce Biotechnology, Rockford, IL). Samples were run on SDS-polyacrylamide gels, transferred onto nitrocellulose membranes, and incubated with appropriate primary antibodies. Blots were then incubated with peroxidase-conjugated secondary antibodies and visualized by enhanced chemiluminescence (Intron).

### Knock down of gene expression

For miR specific for caspase-1 and NLRP3, target sequences were designed using Invitrogen’s RNAi Designer (http://rnaidesigner.lifetechnologies.com/rnaiexpress). The double-stranded DNA oligonucleotides were synthesized and cloned into the parental vector pcDNA6.2-GW/EmGFP-miR (Invitrogen, Carlsbad, CA). The expression cassette for miR was moved into the pENTR/CMV vector and adenovirus was made as previously reported^46^. The miR sequences were as follows: caspase-1, top strand 5′-TGCTGAGAAAGTACTCCTTGAGAGTCGTTTTGGCCACTGACTGACGACTCTCAGAGTACTTTCT-3′ and bottom strand 5′-CCTGAGAAAGTACTCTGAGAGTCGTCAGTCAGTGGCCAAAACGACTCTCAAGGAGTACTTTCTC-3′; NLRP3, top strand 5′-TGCTGATCACAGTGGGATTCGAAACAGTTTTGGCCACTGACTGACTGTTTCGACCCACTGTGAT-3′ and bottom strand 5′-CCTGATCACAGTGGGTCGAAACAGTCAGTCAGTGGCCAAAACTGTTTCGAATCCCACTGTGATC-3′.

### NF-κB inhibition

The IκBα super-repressor has two amino acid substitutions (S32A/S36A) which renders this mutant IκBα resistant to phosphorylation and degradation, thus blocking canonical NF-κB activation. IkBa super-repressor was subcloned into pENTR/CMV-HA vector and adenovirus was made as previously reported^46^.

### Luciferase reporter assay

A NF-κB-luciferase reporter adenovirus was co-transduced with adenoviruses expressing LacZ (control) or IκBα super-repressor (SR) for overnights. After replenishing with fresh medium, cells were cultured for 2 days and then treated with poly(I:C) (10 μg/ml) for 4 hr. Cells were harvested and luciferase activity was measured using the dual luciferase reporter assay system (Promega, Madison, WI).

### Subcellular fractionation

Subcellular fractionation were prepared using NE-PER Nuclear and Cytoplasmic Extraction Reagents (Thermo Scientific, Rockford, IL), according to the recommended protocol. To confirm the purity of subcellular fractionation, the extracts were western blotted and probed with cytoplasmic specific anti-α-tubulin antibody and nuclear specific anti-laminB antibody.

### Statistical analysis

All experiments were repeated at least three times with separate batches of cells. Student *t*-test was used to evaluate statistical significance between two groups. One-way analysis of variance (ANOVA) was used to compare statistical significance within and among three or more groups. Statistical significance was set at p < 0.05.

## Additional Information

**How to cite this article:** Shin, J.-M. *et al*. Double-stranded RNA induces inflammation via the NF-κB pathway and inflammasome activation in the outer root sheath cells of hair follicles. *Sci. Rep.* **7**, 44127; doi: 10.1038/srep44127 (2017).

**Publisher's note:** Springer Nature remains neutral with regard to jurisdictional claims in published maps and institutional affiliations.

## Supplementary Material

Supplementary Information
