# Supplementary material for: Double-stranded RNA induces inflammation via the NF-κB pathway and inflammasome activation in the outer root sheath cells of hair follicles
Source: Sci Rep. 2017 Mar 7;7:44127. doi: 10.1038/srep44127 (PMC5339809; doi:10.1038/srep44127)
Supplement: Supplementary Information [file srep44127-s1.pdf]

Supplementary Information

Double-stranded RNA induces inflammation via the NF-κB pathway and inflammasome activation in the outer root sheath cells of hair follicles

Jung-Min Shin[1], Dae-Kyoung Choi[1], Kyung-Cheol Sohn[1], Soo-Yeon Kim[1], Jeong Min Ha[1], Young Ho Lee[2], Myung Im[1], Young-Joon Seo[1], Chang Deok Kim[1], Jeung-Hoon Lee[1], Young Lee[1*]

[1]Department of Dermatology, School of Medicine, Chungnam National University, Daejeon, Korea
[2]Department of Anatomy, School of Medicine, Chungnam National University, Daejeon, Korea

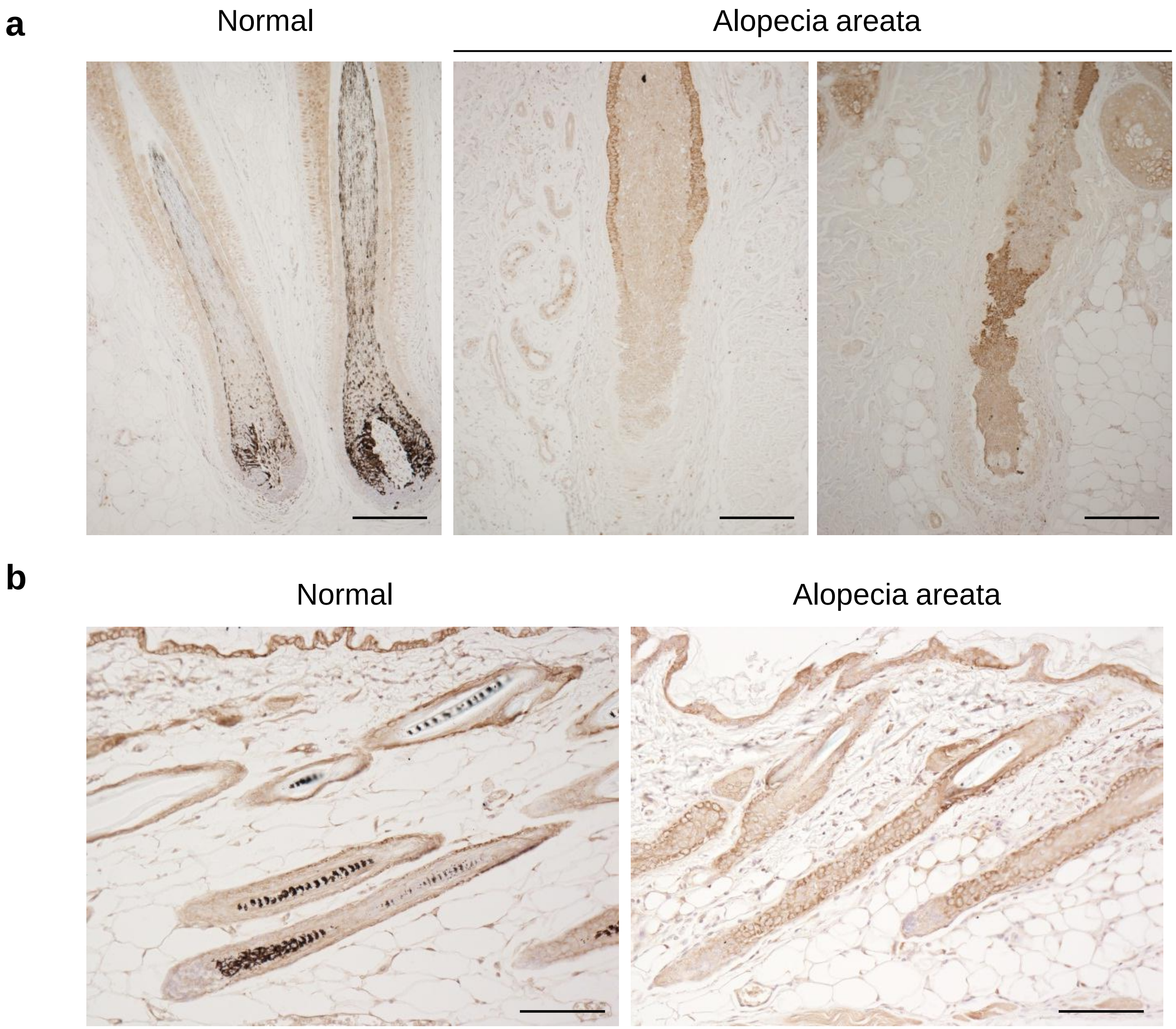

Supplementary Figure 1. Expression of Toll-like receptor 3 (TLR3) in normal and alopecia areata-affected scalp tissues.

Paraffin-embedded normal scalp skin and alopecia areata lesions were immunohistochemically stained with anti-TLR3. Bars = 100 μm.

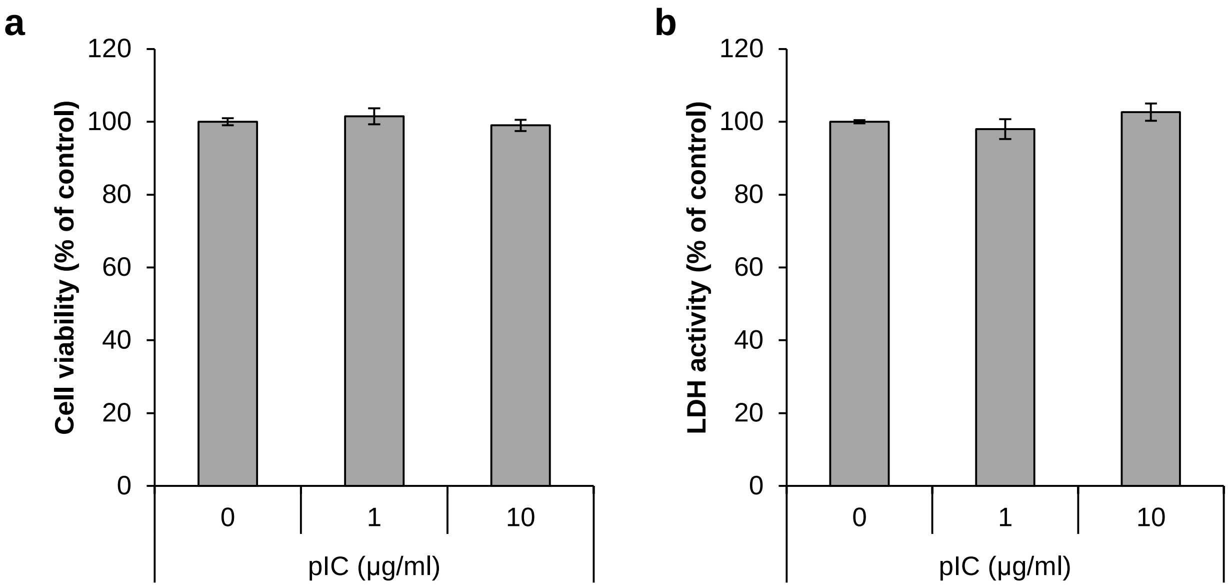

Supplementary Figure 2. Effect of poly(I:C) on cell viability in dermal papilla cells

SV40T-transformed human dermal papilla cells (SV-DP) were treated with poly(I:C) at the indicated concentrations for 24 hr.

(a) Cell viability was measured by MTT assay. (b) Pyroptosis was determined by LDH activity.

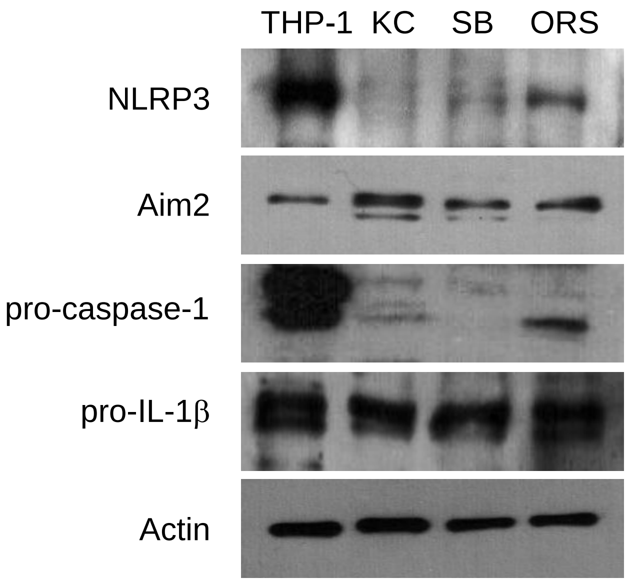

Supplementary Figure 3. Expression of inflammasome components in skin cells

Cell extracts prepared from monocyte-derived THP-1 cell line, normal human epidermal keratinocytes (KC), human sebocytes (SB) and SV40T-transformed human ORS cells (ORS) were assessed by western blotting. Actin was used as a loading control.

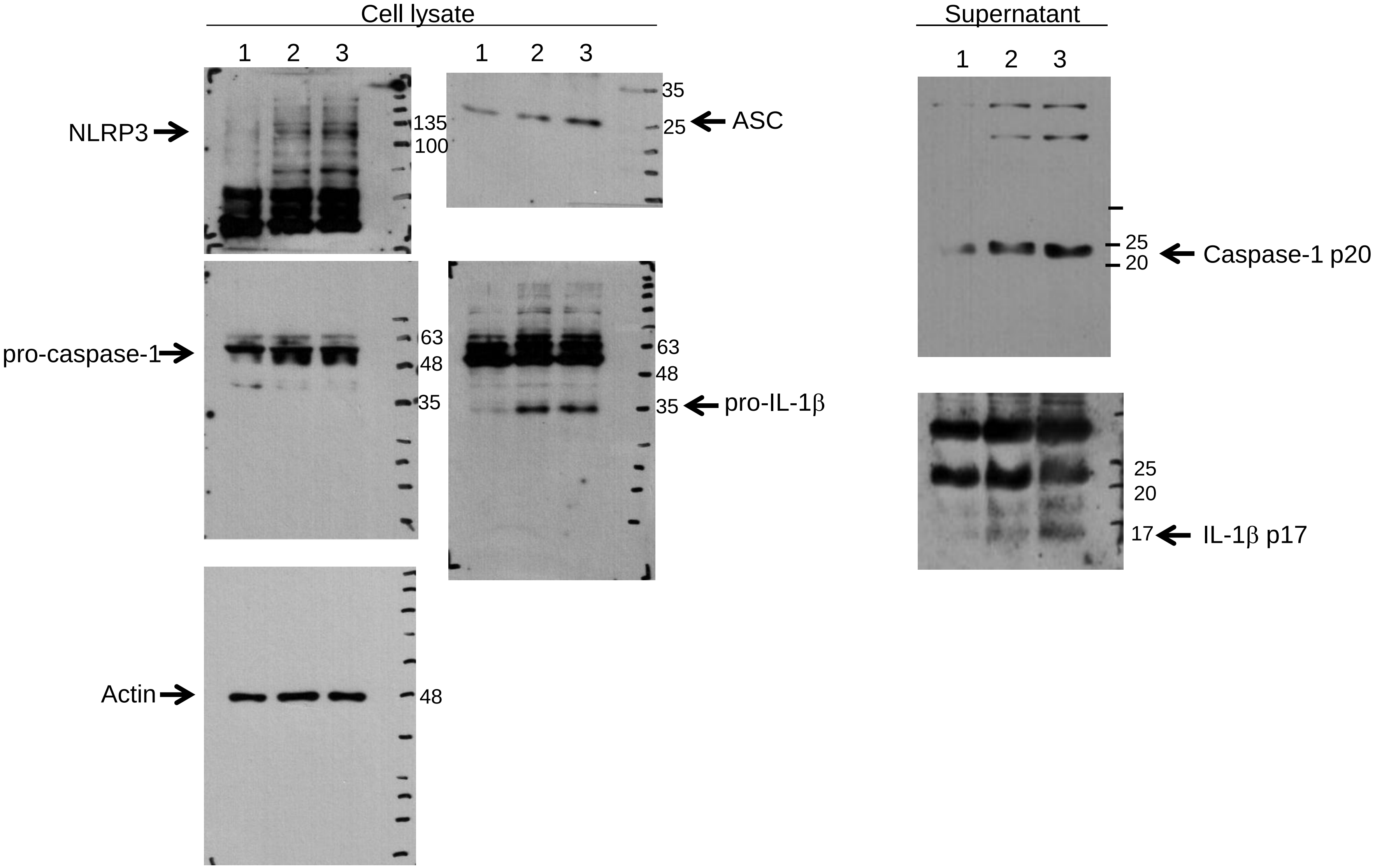

Supplementary Figure 4. Uncropped data for Figure 2e.

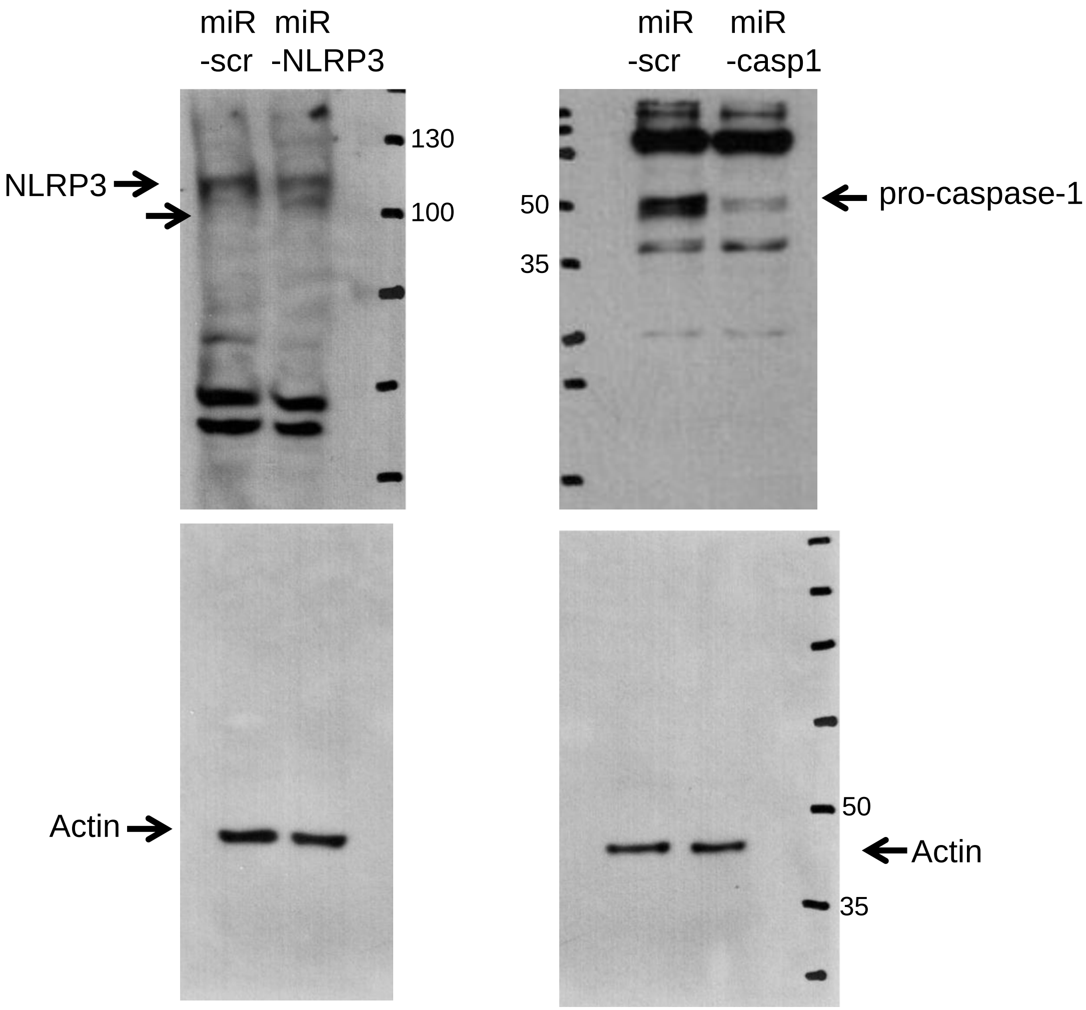

Supplementary Figure 5. Uncropped data for Figure 3b.

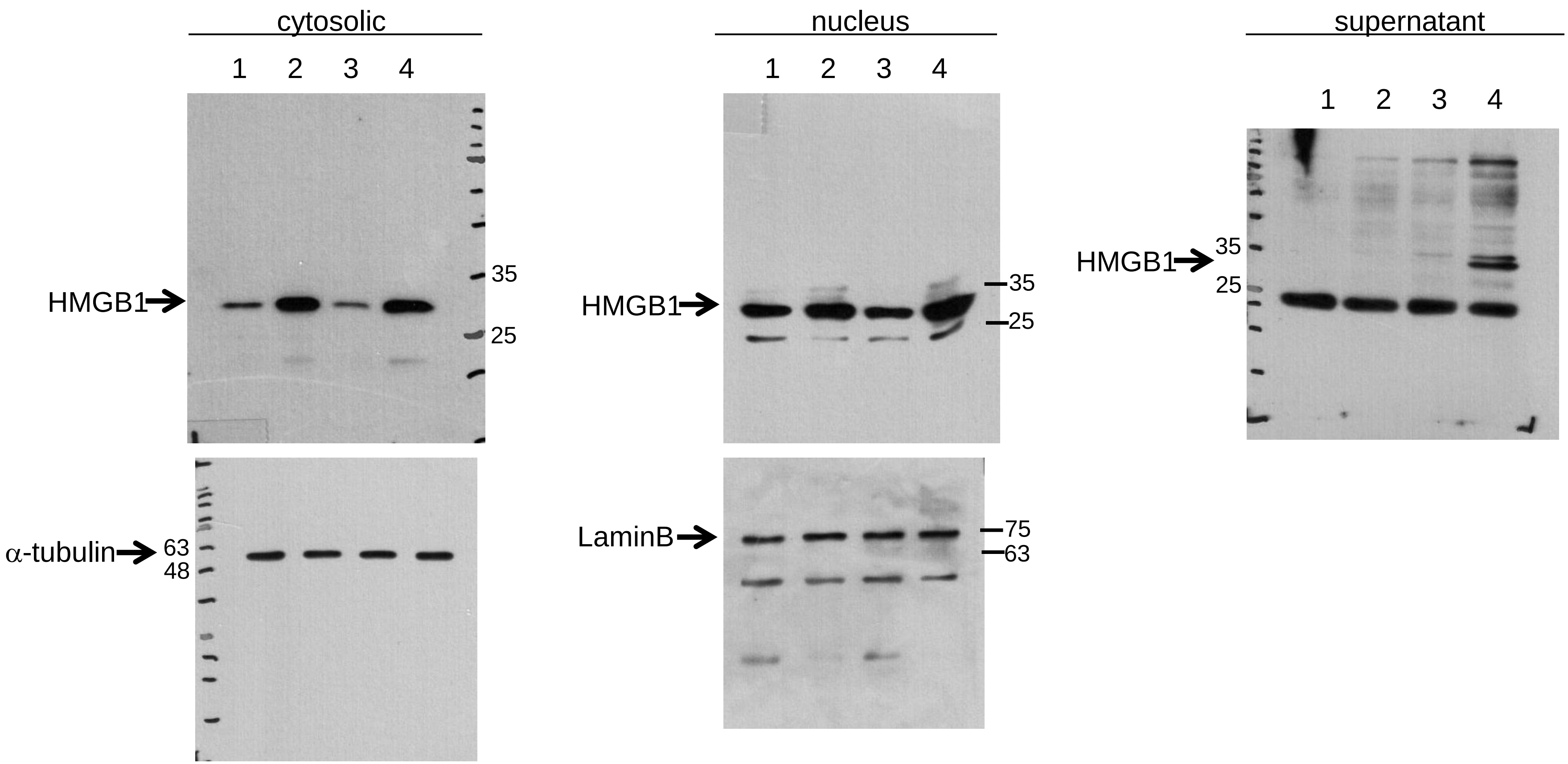

Supplementary Figure 6. Uncropped data for Figure 5a.
